# Improved Diagnostics Using Polarization Imaging and Artificial Neural Networks

**DOI:** 10.1155/2007/74143

**Published:** 2007-11-06

**Authors:** Jianhua Xuan, Uwe Klimach, Hongzhi Zhao, Qiushui Chen, Yingyin Zou, Yue Wang

**Affiliations:** ^1^Department of Electrical and Computer Engineering, Virginia Polytechnic Institute and State University, Arlington, VA 22203, USA; ^2^Department of Oncology, Lombardi Comprehensive Cancer Center, Georgetown University Medical Center, Washington, DC 20057, USA; ^3^Boston Applied Technologies, Inc., Woburn, MA 01801, USA

## Abstract

In recent years, there has been an increasing interest in studying the propagation of polarized light in biological cells and tissues. This paper presents a novel approach to cell or tissue imaging using a full Stokes imaging system with advanced polarization image analysis algorithms for improved diagnostics. The key component of the Stokes imaging system is the electrically tunable retarder, enabling high-speed operation of the system to acquire four intensity images sequentially. From the acquired intensity images, four Stokes vector images can be computed to obtain complete polarization information. Polarization image analysis algorithms are then developed to analyze Stokes polarization images for cell or tissue classification. Specifically, wavelet transforms are first applied to the Stokes components for initial feature analysis and extraction. Artificial neural networks (ANNs) are then used to extract diagnostic features for improved classification and prediction. In this study, phantom experiments have been conducted using a prototyped Stokes polarization imaging device. In particular, several types of phantoms, consisting of polystyrene latex spheres in various diameters, were prepared to simulate different conditions of epidermal layer of skin. The experimental results from phantom studies and a plant cell study show that the classification performance using Stokes images is significantly improved over that using the intensity image only.

## 1. INTRODUCTION

With recent improvements in optical components, the acquisition of polarized images has become easier and more cost effective. Particularly, polarization imaging can reveal important optical properties of the imaged sample in addition to those revealed by a simple intensity imaging method. The fact that the polarization state of the light contains useful information has been shown in many literatures, for example, in [1–3]. Rahmann and Canterakis describe how the polarization state of light can be used for specular surface reconstruction to determine the shape of any three-dimensional (3D) object [1]. They use the fact that light reflected by dielectrics and metals becomes linearly polarized and that the direction of polarization depends on the orientation of the reflecting surface. Demos and Alfano demonstrate a technique based on polarization imaging that allows for optical imaging of a surface as well as structures beneath the surface [2].

The interest of applying polarization imaging to study biological cells or tissues has been shared among many biomedical researchers from very early years to nowadays [4–15]. As early as in l949, it was reported that the activity of nerve cells was associated with changes in their optical properties [4]. When photons impinge on biological materials, their transmission depends on a combination of reflectance, scattering, and absorption effects. Absorption occurs at specific wavelengths, determined by the molecular properties of the materials in the light path. The relatively good transparency of biological materials in the visible and near-infrared (NIR) region of the spectrum permits sufficient photon transmission through organs in site for monitoring cellular events. It has been known for many years that some intrinsic changes in the optical properties of the tissue are dependent on electrical or metabolic activity [5, 6]. Changes in optical properties of brain cells have been reported in cell cultures, brain slices, as well as in intact cortical tissue [7]. Based on assessment of absorption and scattering, three types of activity-related signals have been recorded noninvasively: (1) changes in haemoglobin oxygenation, (2) changes in cytochrome-c-oxidase (co), and (3) optical signals presumably related to changes in light scattering reflecting either membrane potential (fast signals) or cell swelling (slow signal). Villringer and Chance claimed that the advantages of optical methods include biochemical specificity, a temporal resolution in the millisecond range, the potential of measuring intracellular and intravascular events simultaneously, and the portability of the devices enabling bedside examination [8].

The light scattered by a tissue has interacted with the ultrastructure of the biological tissue, which imprinted some intrinsic properties of the tissue. Tissue ultrastructure extends from membranes to membrane aggregates to collagen fibers to nuclei to cells. Photons are most strongly scattered by those structures whose size matches the photon wavelength. It has been demonstrated that light scattering can provide structural and functional information about the tissue [9, 10]. One important biomedical application of optical imaging and spectroscopy is noninvasive or minimally invasive detection of precancerous and early cancerous changes in human epithelium, such as dysplasia or carcinoma in situ.

Recently, many researchers have proposed various optical sensing modalities that could potentially be used to aid in the diagnosis of superficial cancers and other dermatological conditions. In 2000, Jacques et al. demonstrated the use of polarized light for superficial tissue imaging [11]. In their study, they showed that by simply collecting two polarization images through aligned and crossed polarizers and then computing the degree of linear polarization, image contrast can be significantly improved thus revealing superficial structures previously not apparent. In the methods proposed by other researchers, optical polarizers and retarders were varied to provide additional incident and analyzed polarization states, thus enabling the reconstruction of a two-dimensional (2D) Mueller matrix of biological samples [12–15].

This paper presents a new approach to improved cell or tissue classification through the application of Stokes imaging techniques and artificial neural networks [4, 5]. Measuring the polarization of backscattered light provides insight into the optical properties of the cell or tissue, which could lead to improved diagnosis of different tissue types. The polarization state of light can be represented by Jones vectors or by Stokes vectors [16]. In particular, Stokes vectors can represent fully polarized light as well as partially or unpolarized light, hence a natural choice for polarization image representation. In this paper, the intensity images, taken from the backscattered light of the samples, are first converted to Stokes vector images. Note that the Stokes vector, one for each pixel location, fully describes the polarization state of the light at this particular spatial position. In addition, a broadband light source coupled with a tunable optical bandpass filter allows for the illumination and collection of images at different optical wavelengths.

Artificial neural networks (ANNs) have been introduced to classify different cell/tissue types. In particular, multilayer perceptrons (MLPs) are used to extract polarization signatures of the cells or tissues, through which a nonlinear decision boundary can be determined to classify the cells or tissues [17]. To further improve the classification performance, we have also included a feature extraction step using wavelet transforms to derive a joint space and frequency representation of Stokes images [18]. In order to compare the classification performance of Stokes imaging with conventional imaging technique, we have constructed three realistic phantoms using different sizes of polybeans to simulate the epidermal layer of skin. The classification performance, either using full Stokes vector information or using intensity information only, is estimated by a cross-validation method (i.e., 3-fold cross-validation) to demonstrate an improved performance of the proposed polarization imaging system.

The paper is organized as follows. In Section 2, we will describe the polarization imaging system in principle. Specifically, we will describe the design of polarization image device and its image analysis algorithms for classification. In Section 3, we will present a detailed report of preliminary experimental results using phantom studies and a plant cell study, especially on the results of using either single- or multispectral polarization information. Finally, in Section 4 we will conclude this paper with some future research directions.

## 2. METHOD

In this section, we will describe the principle, design, and algorithms of the proposed polarization system in detail. In the design, the polarization imaging device is targeted to acquire a compact representation of polarization information. Technically, a sequential acquisition procedure is proposed to acquire four intensity images with different polarization properties. Four Stokes images are then computed from the intensity images to give a full description of polarization states. In the data analysis subsystem, image analysis algorithms are developed for feature extraction and classification, in which wavelet transforms are used to extract polarization features and artificial neural networks are trained for binary classification.

### 2.1. Polarization imaging device

**Figure 1:**
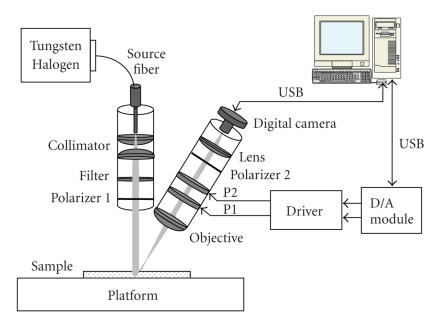
A system diagram of the polarization imaging device.

A detailed diagram of the polarization imaging device is shown in Figure 1. As we can see from the figure, the device is composed of two aluminum cylindrical tubes for illuminator and detector, respectively. A 150-Watt tungsten-halogen lamp is used as the light source providing a strong intensity over a broad spectrum ranging from ultraviolet (UV) (330 nm) to near infrared (NIR) (2 μm). An optical fiber bundle guides the light to the illuminator consisting of a collimator, a 0°-aligned polarizer and a filter. The tunable optical bandpass filter allows us to select a desired illumination wavelength for imaging, ranging from visible light to NIR. The detector consists of an optical objective lens with an infinite focal length, followed by two OptoCeramic (OC) electro-optic phase retarders (with their axes aligned at 45° and 22.5°, respectively), a 0°-aligned linear polarizer, and a digital camera. Two phase retarders are controlled independently by a computer through a two-channel driver, and a D/A module with USB interface. The detector is mounted onto an adjustable station that can perform anglular rotation. Further, a three-dimensional (3D) adjustable platform is used to hold phantoms and other testing samples. Hence, the system is flexible enough to examine the sample with variable incident and collection angles.

**Figure 2:**
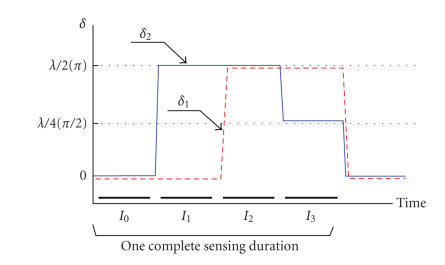
Timing diagram of control voltages applied to the phase retarders OC P1 and OC P2.

In order to acquire the polarization information, that is, the Stokes images, four intensity images ($I_{0}$–$I_{3}$) are taken sequentially, with two phase retarders (i.e., P1 and P2) controlled by a sequence of voltages as shown in Figure 2. Specifically, $I_{0}$ is taken first with 0 volt applied to both P1 and P2; $I_{1}$ is taken second with a half-wave voltage $V_{\pi }$ applied to P1 and 0 volt applied to P2; $I_{2}$ is taken third with $V_{\pi }$ is applied to both P1 and P2; Finally, $I_{3}$ is taken with a quarter-wave voltage $V_{\pi /2}$ applied to P1 and $V_{\pi }$ applied to P2. From the intensity images, that is, $I_{0}$–$I_{3}$, four Stokes vector images $S_{0}$–$S_{3}$ can be calculated by the following equations:

(1) $$\begin{array}{cc} S_{0} & =0.5\times (I_{0}+I_{1}),\\ S_{1} & =0.5\times (I_{0}-I_{1}),\\ S_{2} & =0.5\times (I_{2}-S_{0}),\\ S_{3} & =0.5\times (I_{3}-S_{0}). \end{array}$$

### 2.2. Image analysis algorithms

**Figure 3:**
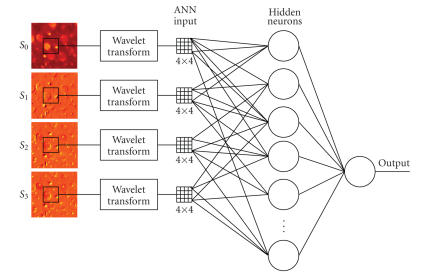
A diagram of image analysis algorithms with wavelet transform (WT) and artificial neural network (ANN).

Figure 3 shows the diagram of the polarization image analysis subsystem. Wavelet transforms (WTs) are applied to Stokes images to extract polarization features; and artificial neural networks (ANNs) are then trained to classify the patterns based on the extracted features. Below we will describe the principle of WTs and ANNs briefly, their application to polarization image analysis, and their performance evaluation based on cross-validation.

#### 2.2.1. Wavelet transform for feature extraction

**Figure 4:**
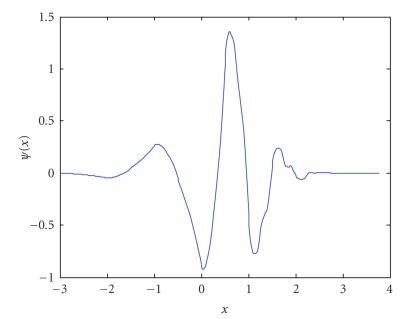
An example of wavelet function-Daubechies D4 function.

Wavelet transforms are introduced to extract polarization features by providing the space and frequency information simultaneously, resulting in a space-frequency representation of the signal [19]. The definition of a continuous wavelet transform for any 1-D signal f(x) can be described as

(2) $$W(a,b)=\frac{1}{\sqrt{a}}\int f(x)\phi^{*}(\frac{x-b}{a})dx,$$

where z^*^ denotes the complex conjugate of *z*, ϕ(x) the analyzing wavelet, *a* the scale parameter, and *b* the position parameter. The wavelet function ϕ(x) can be chosen as simple as the Harr function or one of the popular Daubechies functions. Figure 4 shows the waveform of Daubechies D4 function [6]. By scaling and shifting the wavelet function ϕ(x), we can construct a family of analyzing functions $\phi_{a,b}(x)=\phi (x-b/a)$ to obtain a space-frequency representation of the original signal. In this project, we apply wavelet transforms to polarization images to extract detailed features or signatures for classification of different polarization properties.

**Figure 5:**
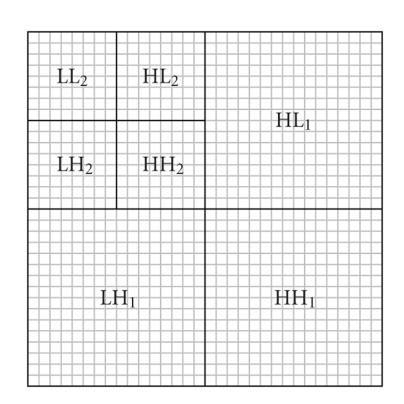
2D wavelet transform-pyramid decomposition of the image.

Using the Haar(or Daubechies D4) function as a transform basis, an image can be decomposed into four separate bands (denoted as LL_1_, LH_1_, HL_1_, and HH_1_; see Figure 5). The LL_1_ band contains a scaled-down, low-resolution version of the original image and the remaining three bands (LH_1_, HL_1_, and HH_1_) contain the detail information (i.e., horizontal, vertical, and diagonal orientation features) about the original image. The process of the wavelet transform can be repeated by transforming the LL_1_ band into a second-level representation: four subbands denoted as LL_2,_LH_2_, HL_2_, and HH_2_. This repeating process, as illustrated in Figure 5, is also called pyramid decomposition [19].

#### 2.2.2. Artificial neural networks for classification

After the feature extraction step, we use multilayer perceptrons (MLPs), a type of nonlinear ANNs, to perform binary classification (see Figure 3). MLPs have been successfully applied to solve a variety of nonlinear classification problems [17]. In our experiments, we specifically develop three-layer perceptron networks for the polarization imaging system. The so-called hidden nodes (neurons) in the middle layer can further extract diagnostic features from the input patterns for nonlinear classification. The connectivity weights are trained or learned in a supervised manner using the error back-propagation algorithm [17].

One of the drawbacks of neural networks is that they do not perform well when the number of inputs is too large (the so-called curse-of-dimensionality phenomenon) [20]. For this reason only the 16 lower band coefficients of the transformed blocks (in a size of 32×32) are selected as inputs to the MLP. Since the neural network receives input values from four component images (16 input values from each component), the total number of inputs to the neural network is 64. The neural network is trained with a standard steepest decent backpropagation algorithm, where its weights are initialized with small randomly selected values. The transfer function for both hidden layer and output layer is the sigmoid function $f\left(x\right)=1/\left(1-e^{-x}\right)$. The target values for two classes are 0 for the first class and 1 for the second class, respectively.

To estimate the generalizable performance of our classification scheme, cross-validation is used to calculate classification error rates (CERs) of the MLP. The input blocks are randomly divided into two sets, one set is used for training and the other is used for cross-validation. This random division of blocks into two sets was repeated 10 times and the neural networks are retrained and tested. The mean classification error rate (CER) and its standard deviation are then computed to evaluate the classification performance. In practice, we performed either L leave-one-out (LOO) test (i.e., holding out one block for testing) or 3-fold cross-validation (i.e., holding out 1/3 of the blocks for testing) to estimate the classification error rates.

To compare the performance of polarization imaging to that of unpolarization imaging, we computed the improvement of polarization imaging over unpolarization imaging using the following formula:

(3) $$\text{Improvement}=\frac{\text{CE}{\text{R}}_{\text{unpolarized}}-\text{CE}{\text{R}}_{\text{polarized}}}{\text{CE}{\text{R}}_{\text{unpolarized}}}\times 100\%,$$

where $\text{CE}{\text{R}}_{\text{polarized}}$ and $\text{CE}{\text{R}}_{\text{unpolarized}}$ are the estimated CERs of polarization imaging and unpolarization imaging, respectively.

## 3. EXPERIMENTAL RESULTS

In this section, we will report our preliminary results of using polarization imaging and artificial neural networks for improved diagnostics. First, the polarization imaging device has been developed using two electrically tunable retarders for acquiring polarization images. Second, different types of phantoms were built to simulate the epidermal layer of the skins for testing the performance of the proposed system. Third, image analysis algorithms have been developed to extract polarization features and classify the phantoms and plant cells. The performance of classification accuracy was evaluated by cross-validation, and the improvement of performance was demonstrated by comparing the performance of the system using polarization information over that without using polarization information.

### 3.1. Polarization imaging system

**Figure 6:**
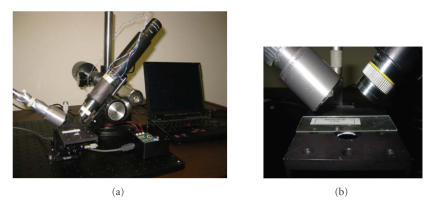
(a) Stokes imaging apparatus consisting of the illuminator and detector. (b) Close-up view of the adjustable platform used to hold the phantom samples.

Figure 6 shows a photograph of the prototyped imaging device, showing an illuminator tube, a detector tube, and an adjustable platform for holding testing samples. The key component, electrically tunable retarder, which supports high-speed operation of the Stokes polarization imaging system, is based on BATi’s newly breakthrough electro-optical ceramic material featuring high electro-optic effect, high operation speed, ruggedness, and ease of fabrication [21]. As described in Section 2, a sequential image acquisition scheme has been implemented to acquire four intensity images with different polarization properties. From our experience, we learn that the accuracy of Stokes polarization imaging is mainly determined by the accuracy of retardation on the phase retarders. Therefore, a careful phase retarder characterization and calibration procedure is developed to minimize the errors between measured and desired phases. In our experiments, the error is less than 0.035 rad, which meets the requirement of the proposed system.

### 3.2. Phantom preparation and data acquisition

The polystyrene phantoms were used to simulate the epidermal layer of the skin. Three phantom samples, Phantom-42, Phanom-74, and Phantom-99, were prepared using polystyrene latex spheres with mean diameters of 42 μm, 74 μm, and 99 μm, respectively. For all 42 μm, 74 μm, and 99 μm spheres, distilled water was added to adjust the reduced scattering coefficient (denoted as $\mu_{\text{s}}$) to match the scattering property of the skin. India ink was also added to latex phantoms to make the absorption coefficient (denoted as $\mu_{\text{a}}$) to match that of the epidermis. The optical properties of the polystyrene phantoms were set at $\mu_{\text{s}}$ = 2.0/mm and $\mu_{\text{a}}$ = 2.46/mm. An Intralipid solid phantom was then used to simulate the dermal layer of the skin. The Intralipid solid phantom was made from agar (a stiffening agent), distilled water, India ink, and 20% Intralipid. The optical properties of the phantom were adjusted to the following numbers: $\mu_{\text{s}}$ = 2.0/mm and $\mu_{\text{a}}$ = 0.03/mm. The optical properties of the polystyrene phantom and Intralipid solid phantom approximated the scattering and absorption of the epidermal and dermal layer of the skin in the range of 550 nm–950 nm. For each sample, a diameter of 3 cm cup of Intralipid solid phantom was placed below the incident light. To simulate a thin skin layer, a small volume of polystyrene phantom was placed onto the center of the solid phantom. This drop spread out in a uniform circle with a diameter that could be easily measured using a Vernier caliper. With the original volume and area covered by the spheres, we can calculate the polystyrene thickness approximately. After waiting a few seconds for the drop area to become stable, polarization images were taken. Here, the thickness was controlled between 50 μm and 170 μm, which is comparable to the thickness of an epidermal layer of the skin (varying from 70 μm to 150 μm for a thin skin).

**Figure 7:**
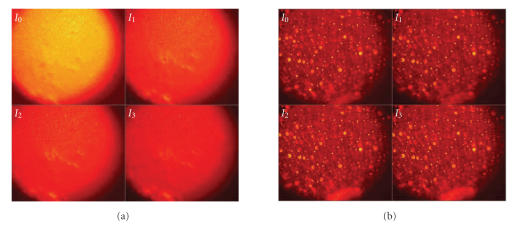
Intensity images ($I_{0}$–$I_{3}$): (a) Phantom-42, and (b) Phantom-99. Note that the images are collected with an incident angle of 22.5° and a collection angle of 45.0°, at the wavelength of 550 nm.

With Phantom-42, Phantom-74, and Phantom-99, different illumination wavelengths were chosen from a range of visible to near IR. In our experiments, we selected 550 nm, 650 nm, and 950 nm for this study. Figure 7 shows examples of the four intensity images ($I_{0}$–$I_{3}$)of Phantom-42 and Phantom-99 collected by the imaging device. These two sets of images were acquired with an incident angle of 22.5° and a collection angle of 45.0°. The wavelength of the incident light was 550 nm.

**Figure 8:**
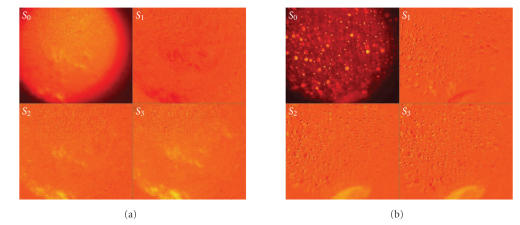
Stokes images ($S_{0}$–$S_{3}$): (a) Phantom-42, and (b) Phantom-99. Note that the images are collected with an incident angle of 22.5° and a collection angle of 45.0°, at the wavelength of 550 nm.

Figure 8 shows the Stokes vector images of Phantom-42 and Phantom-99, respectively, obtained from the intensity images by applying (1). The $S_{0}$ component image reflects the overall intensity (polarized and unpolarized components combined). The component images $S_{1}$, $S_{2}$, and $S_{3}$ contain intensity differences as defined in (1) and can contain positive as well as negative values.

### 3.3. Classification results

With the polarization images acquired using three phantoms (Phantom-42, Phanom-74, and Phantom-99), we have conducted a series of experiments to study characteristics of the polarization system, for example, the incident/collection angle, image quality, and its impact on classification of phantoms. In this section, we will report the classification results on Phantom-42, Phantom-74, and Phantom-99, using either single wavelength or multiple wavelengths of polarization information. The Stokes images were first processed by multiscale wavelet transforms to extract the discriminatory features for classification. The features were then fed to train a three-layer MLP to discriminate two phantoms. To estimate the generalizable classification performance, we used 3-fold cross-validation to compute the mean and standard deviation of the classification error rate. The performance of using polarization information (i.e., using $S_{0}$–$S_{3}$) has been compared to that without using polarization information (i.e., using $I_{0}$ only). From these results we have observed that a significant improvement can be gained by using the polarization information.

Table 1 summarizes the classification performance of various phantom studies with different phantoms (Phantom-42, Phantom-74, and Phantom-99) and different wavelengths (550 nm, 650 nm, and 950 nm). As can be seen, polarization imaging with Stokes information has gained a significant improvement over unpolarization imaging. The improvements on reduction of classification error rate are ranging from 38.27% to 96.82%, resulting in an average improvement of 51.54%. Note that for each case in Table 1, we use the best performances of unpolarized imaging and polarized imaging, respectively, for comparison. Hence, the number of hidden neurons does vary in each case; that is, the ANN classifier is optimized for each case. However, since the performance is not very sensitive to the number of hidden neurons (see Tables 2 and 3 later), we believe that the comparison is reasonable and acceptable for this study.

#### 3.3.1. Polarization imaging study (Phantom-42 versus Phantom-99)

The results presented in this section show the classification performance on polarized phantom images taken at incident wavelengths of 650 nm, 850 nm, and 950 nm, respectively. The network was trained and tested only on images acquired with the same wavelength. In each wavelength category, a total of six images (three for each phantom type) were used. All phantoms were illuminated at an incident angle of 22.5°. The collection angles used were 22.5°, 45.0°, and 67.5°. In order to use 3-fold cross-validation for estimating the network’s classification performance, every image was subdivided into 64 by 64 pixel wide blocks. These blocks were randomly divided into a training set (2/3 of the total number of blocks) and a test set (1/3 of the total number of blocks). From each of these blocks a total number of 81 overlapping windows, 32 by 32 pixels wide, were extracted as inputs to the classification system. The process of dividing the blocks into training and test sets was repeated 10 times to train and test the network. The resulting classification errors were used to calculate the mean classification error and its standard deviation. Tables 2-3 show the results for the wavelengths of 650 nm and 950 nm, respectively.

The classification error rates in the case of the images acquired at 650 nm show an improvement of the classification performance from 18% using intensity only to 11% using Stokes vector images. The error rates in both cases are not dependent on the number of hidden neurons. While the error rate for the training set decreases when more hidden neurons are added (Table 2), the testing error remains constant over a wide range. The standard deviation of the classification error rate is about 2.5%, which is relatively low. In the second case, the incident light wavelength of 950 nm, the classification performance of the Stokes vector images is significantly better than that obtained by using intensity images only. As shown in Table 3, the classification error rate is about 25% when using the intensity information only. When the polarization information is used in addition to the intensity information, the classification error rate decreases to about 16%. Table 3 also shows that the classification error rate is not dependent of the number of hidden neurons either.

#### 3.3.2. Multispectral polarization imaging study (Phantom-42 versus Phantom-74)

The following results show the classification performance on the two phantoms (i.e., Phantom-42 and Phantom-74) using multispectral polarization information. While the results in Section 3.3.1 show the classification performance of the system when trained and tested at a single wavelength, the results in this section show the results when images taken at different wavelengths are combined to train the neural network for classification. Specifically, the images used in this section were taken at the wavelengths of 550 nm, 650 nm, and 950 nm, respectively. As described in the previous section, 3-fold cross-validation was used to estimate the MLP’s classification performance. Again, each image was subdivided into 64 by 64 pixel wide blocks. These blocks were randomly divided into a training set and a test set. From each of these blocks a total number of 81 overlapping windows, 32 by 32 pixels wide, were extracted as inputs to the classification system. The process of dividing the blocks into training and validation set was repeated 10 times. The network was retrained and the classification performance was estimated using the test set. The mean classification error (from cross-validation) and its standard deviation are shown in Table 4 where the number of hidden neurons varies from 1 to 10. As we can see, the classification error rate decreases when additional hidden neurons are added to the neural network. This is the case for using the intensity image ($I_{0}$) only as well as for using four Stokes images ($S_{0}$–$S_{3}$). The network performs significantly better when trained on the Stokes images (7.2% error rate with $S_{0}$–$S_{3}$) than that using intensity image only (24.7% error rate with $I_{0}$ only). It can also be clearly seen from Table 4 that an increase of the number of hidden neurons improves the classification performance. From this limited preliminary study, it seems that the classification problem appears to be more complex when images taken at different wavelengths are combined, and the ANN classifier with more hidden neurons might help extract polarization information for better classification.

#### 3.3.3. Polarization imaging study of plant cells

**Figure 9:**
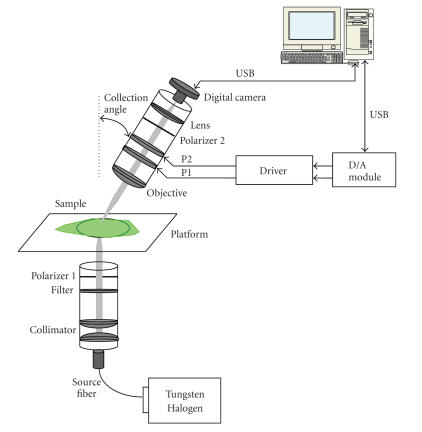
A system diagram of the transmission-mode polarization imaging device.

In this experiment, we collected two kinds of leaves to test our polarization imaging scheme. Two kinds of leaves were picked off from different bushes. Alcohol was used to clean the leave surfaces to get rid of the dusts and other containments. It is known that the plant cells can be viewed or sensed directly by collecting the transmission light. The transmission Stokes imaging system shown in Figure 9 was used for the experiment. The setup shown in Figure 1 was modified to collect the transmission information, as shown in Figure 9. The working principle of the system is same, as that described in Section 2.1, except that the signal would be collected after the light passing through the sample. For those relatively thin and transparent samples, measurement with a transmission mode would be better than with reflective mode in achieving higher signal to noise ratio.

**Figure 10:**
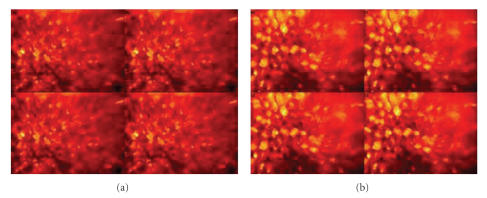
A polarization imaging study of plan cells-original acquired images ($I_{0}$–$I_{3}$): (a) Leaf A and (b) Leaf B.

**Figure 11:**
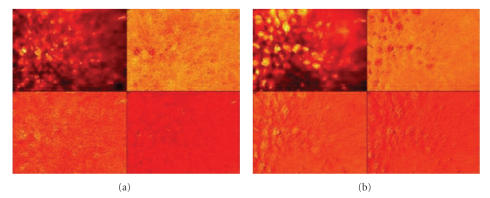
A polarization imaging study of plan cells-stokes images ($S_{0}$–$S_{3}$): (a) Leaf A and (b) Leaf B.

As shown in Figure 10, the $I_{0}$–$I_{3}$ images of Leaf A and Leaf B were acquired using the transmission mode as described above. Figure 11 shows the Stokes images ($S_{0}$–$S_{3}$) of the acquired polarization information, which clearly de-correlate the dependency between $I_{0}$–$I_{3}$. Further processed by wavelet transform and ANN training, the discriminatory features from polarization imaging have been fully captured in hidden neurons for classification. The classification performance is detailed in Table 5, where the improvement over unpolarization imaging is also calculated. The classification error rate (CER) has been significantly reduced from 34.80% for unpolarization imaging to 11.3% for polarization imaging, resulting in an improvement of 64.04% in average.

## 4. CONCLUSION

In this paper, we have presented a polarization imaging device to acquire a complete set of Stokes vector images for improved diagnostics. An image analysis subsystem has also been developed to classify different types of phantoms based on the 2D discrete wavelet transforms (2D-DWTs) and multilayer perceptrons (MLPs). When trained and tested with the complete set of Stokes images (i.e., using $S_{0}$–$S_{3}$), the classification performance is significantly improved compared to that with intensity image only (i.e., using $I_{0}$ only). The results have confirmed that the polarization state contains important information that can be used to classify two different types of phantoms. While the results are encouraging and this study shows the potential of this imaging device, further study is needed. Future work may include more realistic phantom studies and biological cell and tissue studies for validation. Currently, polarization imaging of cell study has been under investigation in collaboration with (Georgetown University Medical Center DC, USA). Some optimization procedure for ANNs (such as the optimized MLP in [22]) will be explored to avoid the local minima problem existed in nonlinear classification problem. As also shown in our preliminary result in Section 3, the use of multispectral polarized images is another possible path to improve the classification performance. Note that although we are encouraged by the initial results using multispectral polarization imaging, we have also realized that the problem seems to be complicated by many other factors, like the proper wavelength and illumination angle selection. To tackle this problem, we believe that some optimization approach needs to be developed in future to replace the current simple combining approach.

## Figures and Tables

**Table 1:** A summary of the classification performance in phantom studies. The average improvement of polarization imaging over unpolarization imaging is 51.54%.

No.	Experiment (Phantom study)	Classification error rate (CER)				Improvement
		Unpolarized (using $I_{0}$)		Polarized (using $S_{0}$–$S_{3}$)		(polarized over unpolarized)
		Mean	Standard deviation	Mean	Standard deviation	
1	Phantom-42 versus Phantom-74 @ 550 nm	15.70%	4.96%	0.50%	0.81%	96.82%
2	Phantom-42 versus Phantom-74 @ 950 nm	17.20%	3.35%	5.70%	2.50%	66.86%
3	Phantom-42 versus Phantom-99 @ 650 nm	17.90%	2.10%	10.30%	1.90%	42.46%
4	Phantom-42 versus Phantom-99 @ 950 nm	24.30%	2.50%	15%	1.70%	38.27%

**Table 2:** Classification error rates obtained with 3-fold cross-validation (Phantom-42 versus Phantom-99). The wavelength of the illuminating light is 650 nm, and the incident angle is 22.5°. Collection angles used are 22.5°, 45.0°, and 67.5°, respectively.

No. of hidden neurons	Classification error rate (CER)				Improvement (polarized over unpolarized)
	Unpolarized (using $I_{0}$)		Polarized (using $S_{0}$–$S_{3}$)		
	Mean	Standard deviation	Mean	Standard deviation	
5	20.00%	2.40%	11.20%	2.40%	44.00%
10	18.40%	2.10%	10.50%	1.90%	42.93%
15	18.30%	2.50%	10.90%	1.80%	40.44%
20	18.00%	2.00%	10.80%	2.10%	40.00%
25	18.00%	2.10%	10.40%	2.20%	42.22%
30	17.90%	2.10%	10.30%	1.90%	42.46%

**Table 3:** Classification error rates obtained with 3-fold cross (validation – Phantom-42 versus Phantom-99). The wavelength of the illuminating light is 950 nm, and the incident angle is 22.5°. Collection angles used are 22.5°, 45.0°, and 67.5°, respectively.

No. of hidden neurons	Classification error rate (CER)				Improvement (polarized over unpolarized)
	Unpolarized (using $I_{0}$)		Polarized (using $S_{0}$–$S_{3}$)		
	Mean	Standard deviation	Mean	Standard deviation	
5	24.30%	2.50%	18.10%	2.00%	25.51%
10	25.20%	3.90%	15.90%	1.20%	36.90%
15	25.20%	5.50%	16.90%	2.00%	33.73%
20	27.40%	6.50%	15.00%	1.70%	45.26%
25	24.40%	6.20%	16.70%	0.80%	31.56%

**Table 4:** Multispectral polarization imaging study-classification error rate obtained with 3-fold cross-validation (Phantom-42 versus Phantom 74). The wavelengths of the illuminating light ware 550 nm, 650 nm, and 950 nm, respectively. The incident angle is 22.5°, and the collection angles are 22.5°, 45.0°, and 67.5°, respectively.

No. of hidden neurons	Classification error rate (CER)				Improvement (polarized over unpolarized)
	Unpolarized (using $I_{0}$)		Polarized (using $S_{0}$–$S_{3}$)		
	Mean	Standard deviation	Mean	Standard deviation	
1	44.00%	4.40%	25.20%	3.10%	42.73%
2	37.50%	7.90%	19.00%	6.00%	49.33%
5	28.80%	7.80%	8.70%	4.00%	69.79%
10	24.70%	2.80%	7.20%	1.60%	70.85%

**Table 5:** Classification performance of plant cell studies. The average improvement of polarization imaging over unpolarization imaging is 64.06%.

No. of hidden neurons	Classification error rate (CER)				Improvement (polarized over unpolarized)
	Unpolarized (using $I_{0}$)		Polarized (using $S_{0}$–$S_{3}$)		
	mean	standard deviation	mean	standard deviation	
5	34.80%	4.20%	15.50%	4.50%	55.46%
10	34.30%	4.50%	13.40%	3.30%	60.93%
15	35.50%	3.80%	11.30%	3.20%	68.17%
20	35.30%	3%	11.60%	3.10%	67.14%
25	35.90%	2.90%	11.70%	2.70%	67.41%
30	35.70%	4%	12.40%	2.70%	65.27%
